# Robust synthesis of 2′-azido modified RNA from 2′-amino precursors by diazotransfer reaction†

**DOI:** 10.1039/d2ob01560a

**Published:** 2022-09-29

**Authors:** Sarah Moreno, José M. Ramos Pittol, Markus Hartl, Ronald Micura

**Affiliations:** Institute of Organic Chemistry, Center for Molecular Biosciences Innsbruck, University of Innsbruck Innrain 80-82 6020 Innsbruck Austria ronald.micura@uibk.ac.at; Institute of Biochemistry, Center for Chemistry and Biomedicine (CCB) Innsbruck, University of Innsbruck Innrain 80-82 6020 Innsbruck Austria

## Abstract

Azides are versatile bioorthogonal reporter moieties that are commonly used for site-specific labeling and functionalization of RNA to probe its biology. The preparation of azido modified nucleic acids by solid-phase synthesis is problematic due to the inherent reactivity of P(iii) species with azides according to the Staudinger reaction. Various strategies have been developed to bypass this limitation and are often time-consuming, low-yielding and labor-intensive. In particular, the synthesis of RNA with internal 2′-azido modifications is restricted to a single approach that employs P(v) chemistry instead of the widely used P(iii) phosphoramidite chemistry. To fill this methodological gap, we present a novel convenient path toward 2′-azido RNA from readily accessible 2′-amino RNA through treatment with the diazotizing reagent fluorosulfuryl azide (FSO_2_N_3_). A diazotransfer reaction was established for oligoribonucleotides of different lengths and secondary structures. The robustness of the approach was further demonstrated for RNAs containing multiple 2′-azido moieties and for RNAs containing other sensitive modifications such as thiouridine or methylated nucleobases with a positive charge. The synthetic ease of generating 2′-azido RNA will pave the way for biotechnological applications, in particular for siRNA technologies and for referencing the growing number of RNA metabolic labeling approaches that rely on 2′-azido nucleosides.

## Abstract 2

One-step conversion chemistry makes RNA with internal site-specific 2′-azido modifications readily accessible from 2′-amino RNA; it is significantly more efficient compared to thus far used phosphotriester chemistry to generate such RNA.

## Graphical abstract

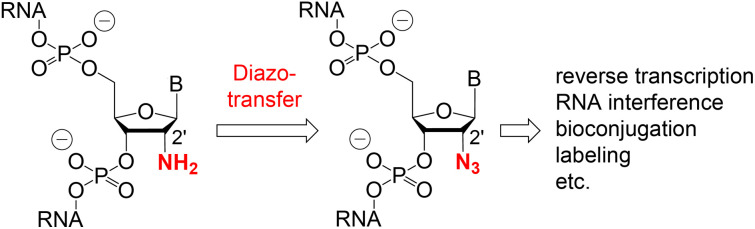

## Introduction

Azides are small and stable modifications that are rarely encountered in living organisms.^1,2^ They do not show any reactivity towards endogenous functional groups and are chemically engineered into the biomolecule of interest.^3,4^ Thus, azido modified RNA is a substantial tool for site-specific labeling and functionalization of RNA to probe its structure, dynamics, and localization, with applications in diagnostics, forensics, genetic analysis and sequencing.^5,6^ In all these approaches, a covalent linkage of the azido group to a reporter molecule, fluorescent dye, affinity tag or transporter unit is formed by either Staudinger ligation, Cu(i)-catalyzed azide–alkyne cycloaddition (CuAAC), strain-promoted [3 + 2] cycloaddition (SPAAC), or photo-click chemistry, with the RNA azido group being located at the ribose, the termini, the phosphate backbone or the nucleobase.^4–9^

For chemical biology, 2′-azido modified RNA is an upcoming tool whose physicochemical properties have been reported earlier.^10–12^ The 2′-N_3_ modification favours the RNA C3′-*endo* sugar pucker, only causes a slight decrease in base pairing stabilities, and hardly influences the overall RNA structure.^10,11^ It is exceptionally well tolerated in the guide strand of siRNAs even when it is directly located at the cleavage site.^10,11^ This and other emerging applications in chemical biology require easy accessibility, *i.e.* the efficient synthesis of RNAs with site-specific 2′-N_3_ anchors. However, the chemical synthesis of azido modified oligonucleotides currently suffers from severe limitations since azides exhibit pronounced reactivity toward P(iii) species (such as nucleoside phosphoramidite building blocks) according to Staudinger-type reactions.^10–17^

**Fig. 1:**
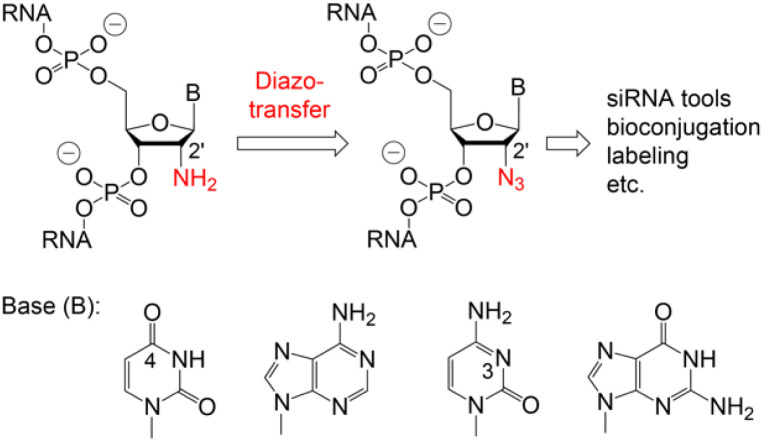
Proposed conversion of 2′-amino into 2′-azido RNA using mild reagent mediated diazotransfer reactions.

Various chemical and biotechnological strategies have been developed to bypass this restriction and strongly depend on the position of the azide modification. To date, the only option for the chemical synthesis of internally 2′-N_3_ modified RNA is provided by the integration of phospho(V)diester building blocks which are manually incorporated by a single coupling cycle of the phosphotriester method during otherwise automated standard oligonucleotide synthesis.^10,11,17^ Consequently, an expansion of the methodological repertoire for efficient and flexible access to 2′-N_3_ modified RNA is urgently needed. Therefore, we developed a convenient path starting from readily available 2′-NH_2_ modified RNA which is converted into 2′-N_3_ RNA by a diazotransfer reaction (Fig. 1).

## Results and discussion

### Diazotransfer on oligonucleotides

The potential of diazotransfer reactions on aminoalkylated DNA was first reported by Defrancq and coworkers^18^ in 2011 by the use of imidazole-1-sulfonylazide hydrochloride (ISAHC) in the presence of divalent metal ions. In 2020, Meng *et al.*^19^ demonstrated the rapid and quantitative conversion of a variety of small, organic amines into their corresponding azides in a biphasic solvent system containing potassium bicarbonate. Remarkably, the reaction requires only one equivalent of fluorosulfuryl azide (FSO_2_N_3_) and was deemed complete within 5 min at ambient temperature for most of the substrates. Shortly after, Krasheninina *et al.*^20^ demonstrated the applicability of FSO_2_N_3_ on native RNA and RNA–peptide conjugates containing an aliphatic primary amino group. The reaction conditions were found to be rather similar with the exception that full conversion required two equivalents of FSO_2_N_3_ and longer reaction times. To further expand the scope of substrates and to enhance the methodological repertoire specifically for the synthesis of 2′-N_3_ modified RNAs, the conversion of 2′-NH_2_-RNAs into their corresponding 2′-N_3_ analogues has been envisioned here. Because the 2′-position is much more sterically hindered than that in all the previously reported substrates, FSO_2_N_3_ may however not be able to approach the reaction centre sufficiently.

### Conversion of 2′-NH_2_ RNA into 2′-N_3_ RNA

The canonical 2′-NH_2_ nucleoside phosphoramidites were obtained from commercial sources or were synthesized in-house;^21–23^ the corresponding 2′-NH_2_ modified RNA was prepared using standard solid-phase synthesis and deprotection protocols.

**Fig. 2:**
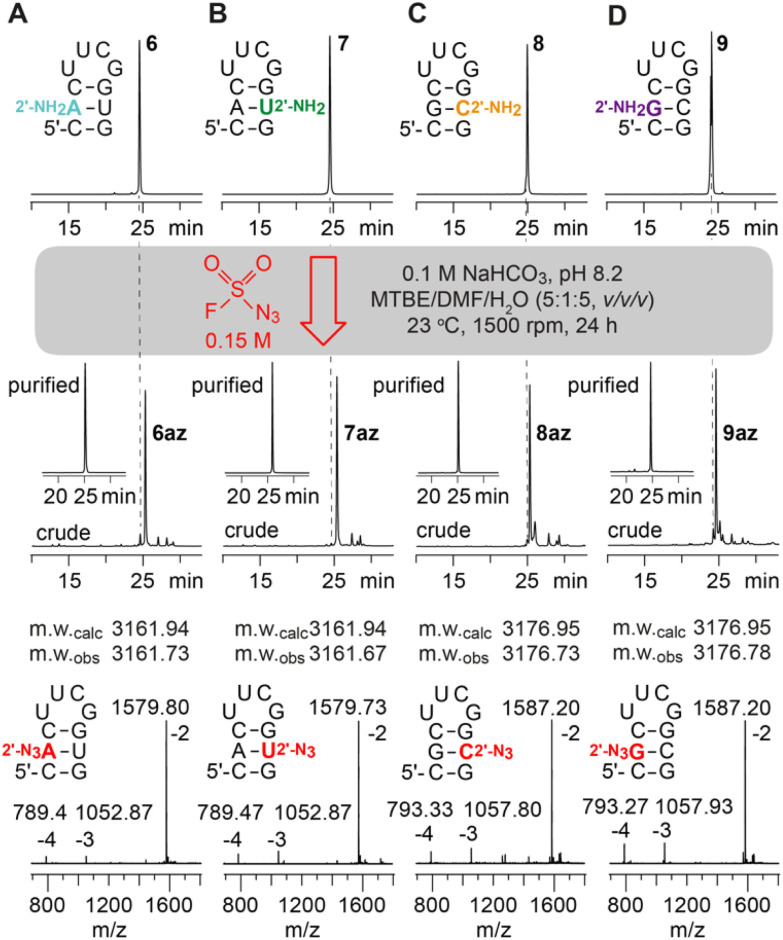
Diazotransfer reaction on a (A) 2′-NH_2_-A; (B) 2′-NH_2_-U; (C) 2′-NH_2_-C; (D) 2′-NH_2_-G modified 10 nt hairpin using 100 μl of FSO_2_N_3_ (150 mM) for 24 h. AE-HPLC trace of the 2′-NH_2_ starting materials (top), crude and purified 2′-N_3_ reaction products (middle), and ESI-mass spectra of purified products (bottom) are shown.

Incubation of the 2′-NH_2_ RNA precursor 1 with FSO_2_N_3_ following the conditions described by Krasheninina *et al.*,^20^ however, did not lead to any product formation. Only when significantly longer reaction times and increased concentration of the diazotizing reagent were used, we achieved complete conversion (ESI Fig. 1–5†). Thereby, the 5 nt long RNA (2–5) without any secondary structure (single strand) showed much faster reaction progress than the 10 nt hairpin (6–9) or the 8 nt palindrome (1, 10–12) that exhibits defined secondary structures (double helices). A summary of the diazotransfer on the 10 nt hairpin for all canonical 2′-NH_2_ modified RNAs (6–9) is shown in Fig. 2. In short, 100 μl of 150 mM FSO_2_N_3_ in methyl *tert*-butyl ether (MTBE) and 24 h reaction time were found to be suitable to achieve full conversion.

To further analyse the structure dependency and to apply the diazotransfer reaction to longer sequences, the 27 nt sarcin-ricin loop^24^ was synthesized and a single 2′-NH_2_-A was placed in the middle of the stem (13), in the loop region (14) or at the 5′-terminus (15) (ESI Fig. 6†). As expected, the loop modified sarcin-ricin structure showed the fastest reaction progress followed by the terminally modified RNA and the RNA where the modification resides in the Watson–Crick paired region. This is consistent with the initial observations on 2′ accessibility.

**Fig. 3:**
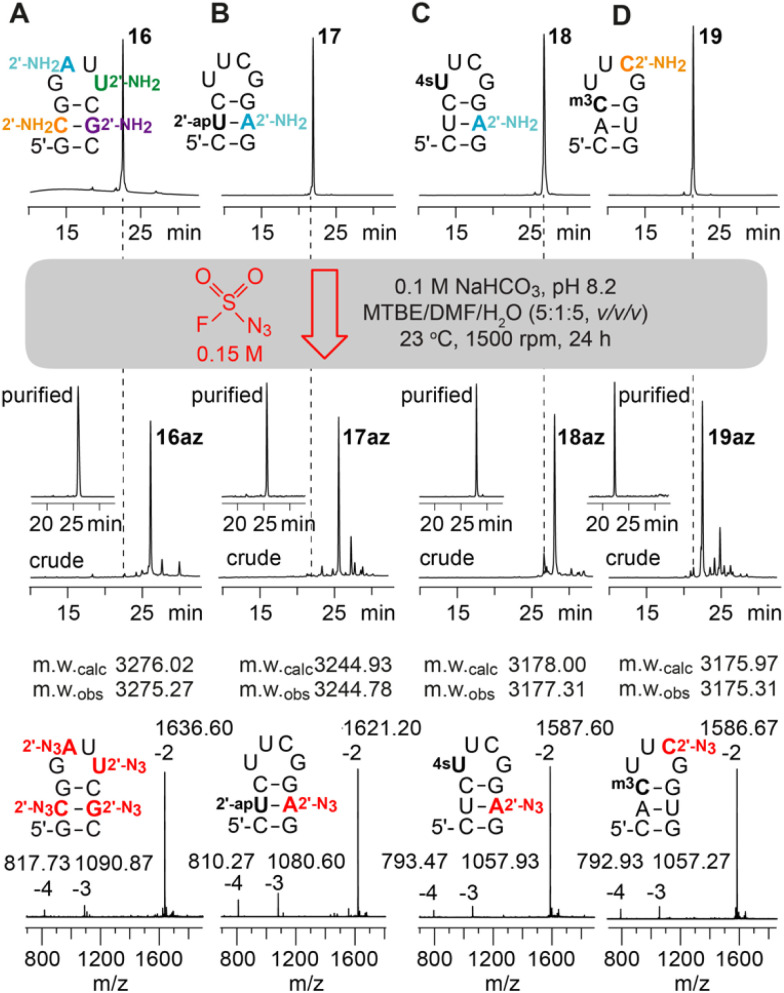
Diazotransfer reaction on a 10 nt hairpin containing (A) all 2′-NH_2_ modified canonical nucleotides; (B) 2′-(3-aminopropyl)uridine (2′-apU) and 2′-NH_2_-A; (C) 2′-NH_2_-A and 4-thiouridine (4sU); (D) 2′-NH_2_-C and 3-methylcytidine (m^3^C). Anion-exchange (AE)-HPLC traces of the starting materials (top), crude and purified 2′-N_3_ reaction products (middle), and the ESI-mass spectra of purified products (bottom).

Next, RNAs containing more than one aliphatic amino group were synthesized and their amenability for multiple diazotransfer was tested. Hence, the 10 nt hairpin (16) was equipped with all four standard 2′-NH_2_ nucleotides (A, C, G, U) at once, and a second hairpin (17) was prepared forming a base pair between 2′-NH_2_-A and 2′-*O*-(3-aminopropyl)uridine.^12,13,25^ The modified RNAs were treated with FSO_2_N_3_ for 24 h and clean conversions for the sequences were observed (Fig. 3A and B).

Subsequently, the compatibility of the diazotransfer reaction with other sensitive and reactive RNA modifications like 4-thiouridine (4sU) and 3-methylcytidine (m^3^C) was tested (18, 19) (Fig. 3C and D). The corresponding phosphoramidite building blocks were synthesized according to published procedures^26,27^ and the modified RNAs were prepared as described in the ESI.† 4sU is one of the most widely applied labels on RNA due to its broad and selective reactivity scope (disulfide formation, oxidative alkylations, photo-crosslinking, *etc*.) and used *e.g.* for biotinylation and RNA pull-down, covalent attachment to fluorophores, or photoactivatable-ribonucleoside-enhanced crosslinking and immunoprecipitation (PAR-CLIP).^28–31^ Moreover, metabolic labeling-based RNA sequencing methods (*e.g.* Thiouridine-to-Cytidine Conversion Sequencing, TUC-seq) have emerged which became indispensable tools for analysing cellular RNA dynamics.^26^ The 4sU modification, however, can also undergo undesired side reactions like oxidation, dimerization, hydrolysis and degradation. Selective derivatization of 4sU modified RNA that leaves the sensitive 4-thio moiety intact is therefore rare. We show that the treatment of 4sU containing RNA (18) with FSO_2_N_3_ indeed results in the selective formation of the desired 2′-N_3_ modified RNA (18az) preserving the parent 4sU modification. The obtained 4sU and 2′-N_3_ modified RNA offers two orthogonal conjugation sites to combine the novel RNA sequencing approaches with efficient bioconjugation reactions.

Next, we focused on testing the diazotransfer reaction for RNA (19) containing 3-methylcytidine (m^3^C) which is another fragile modification due to its positively charged nucleobase. In tRNA, m^3^C has long been known,^32,33^ but recently this modification gained increasing attention due to its evidential occurrence in the mRNA of mice and humans.^34^ Furthermore, the self-methylation activity of a naturally occurring riboswitch scaffold^35^ and various approaches for detection and genome-wide m^3^C sequencing^36–38^ have been reported. Although m^3^C is prone to nucleobase loss (depyrimidization) and/or hydrolysis to 3-methyluridine (see ref. 35), the diazotransfer reaction proceeded smoothly in our hands for the m^3^C and 2′-NH_2_-C modified RNA, furnishing the m^3^C containing 2′-N_3_ analog (19az) in good yields.

Taken together, we have demonstrated that 2′-amino RNAs can be converted into their 2′-azido counterparts by the diazotransfer reaction (see ESI Table 1† for a complete list) in nearly quantitative yields for short RNAs and yields of more than 80 to 90% for longer RNAs. Although extended reaction times (>12 h) are needed, RNA degradation appears minimal under the biphasic reaction conditions, reflected in HPLC traces with no or only a few faster migrating RNA side products. Interestingly, slower migrating side products are observed (less than 10 to 20%) that may indicate covalent RNA-reagent intermediates or even crosslinked RNA dimers.

### Reverse transcription of 2′-NH_2_ and 2′-N_3_ RNA

**Fig. 4:**
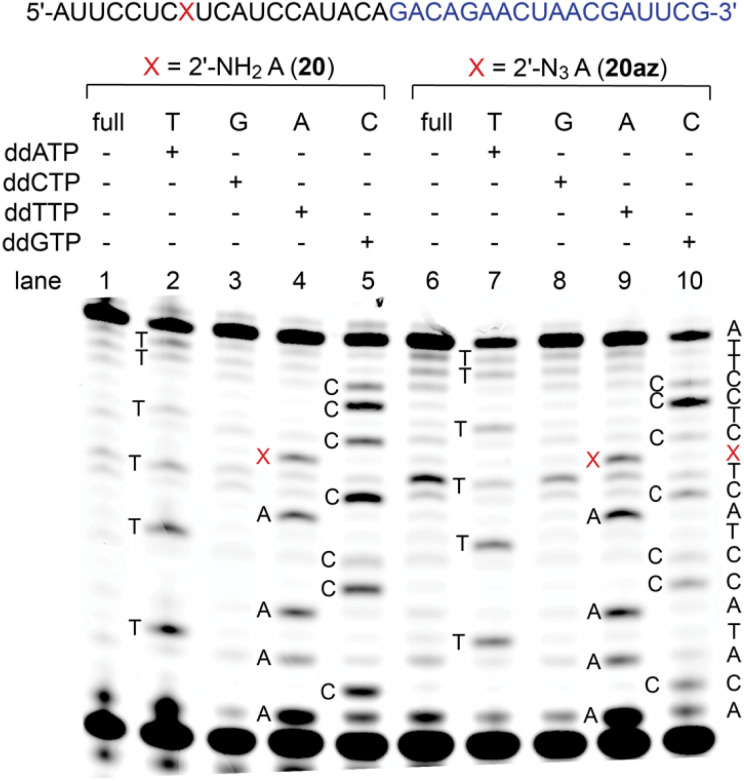
Primer extension assay using SuperScript IV reverse transcriptase for 2′-NH_2_-A (lanes 1–5) and 2′-N_3_-A (lanes 6–10) RNA with the corresponding sequencing ladders. Primer sequence is indicated in blue and the position of the modification is marked as red X. Both modifications are recognized as adenosine. The 2′-N_3_ modification causes slightly enhanced termination of reversed transcription compared to 2′-NH_2_ (lane 1 and 6).

2′-Amino and 2′-azido nucleotide triphosphates (NTPs) are only modestly accepted substrates for native RNA polymerases; however, polymerase mutants have been described that make such NTPs effective for enzymatic RNA synthesis.^39–41^ Here, to shed light on the performance of 2′-NH_2_ and 2′-N_3_ modified RNA templates in reverse transcription, we performed primer extension using SuperScript IV reverse transcriptase on synthetic 37 nt long RNAs (20, 20az) containing either a single 2′-NH_2_- or a single 2′-N_3_ adenosine at the same internal position, flanked by 5′-C and 3′-U (Fig. 4 and ESI Fig. 7, 8†). Both modifications, 2′-NH_2_-A and 2′-N_3_-A, were well recognized by thymidine, and hence, they preserved canonical base recognition. However, slightly enhanced termination of reverse transcription at the 2′-N_3_ modification (Fig. 4, lane 6 and 9) was observed while this was not then case for the 2′-NH_2_ modification (Fig. 4, lane 1 and 4). This observation may be attributed to different preferences of the modified ribose pucker (C3′- *versus* C2′ *endo* conformation) and/or to the larger steric hindrance of 2′-N_3_ compared to that of the 2′-NH_2_ group.

### Potential of 2′-NH_2_ and 2′-N_3_ siRNAs

Earlier on, we have pointed out the promising potential of 2′-azido modified RNA for RNA interference,^10,11^ being equivalent or even superior to 2′-F and 2′-OCH_3_ modified siRNAs that have found biomedical and therapeutic applications.^42,43^ As shown previously, single 2′-N_3_ moieties were excellently tolerated in the guide strand of the siRNA duplex and caused efficient gene silencing even when the modification was located in the seed region or at the cleavage site.^10,11^

**Fig. 5:**
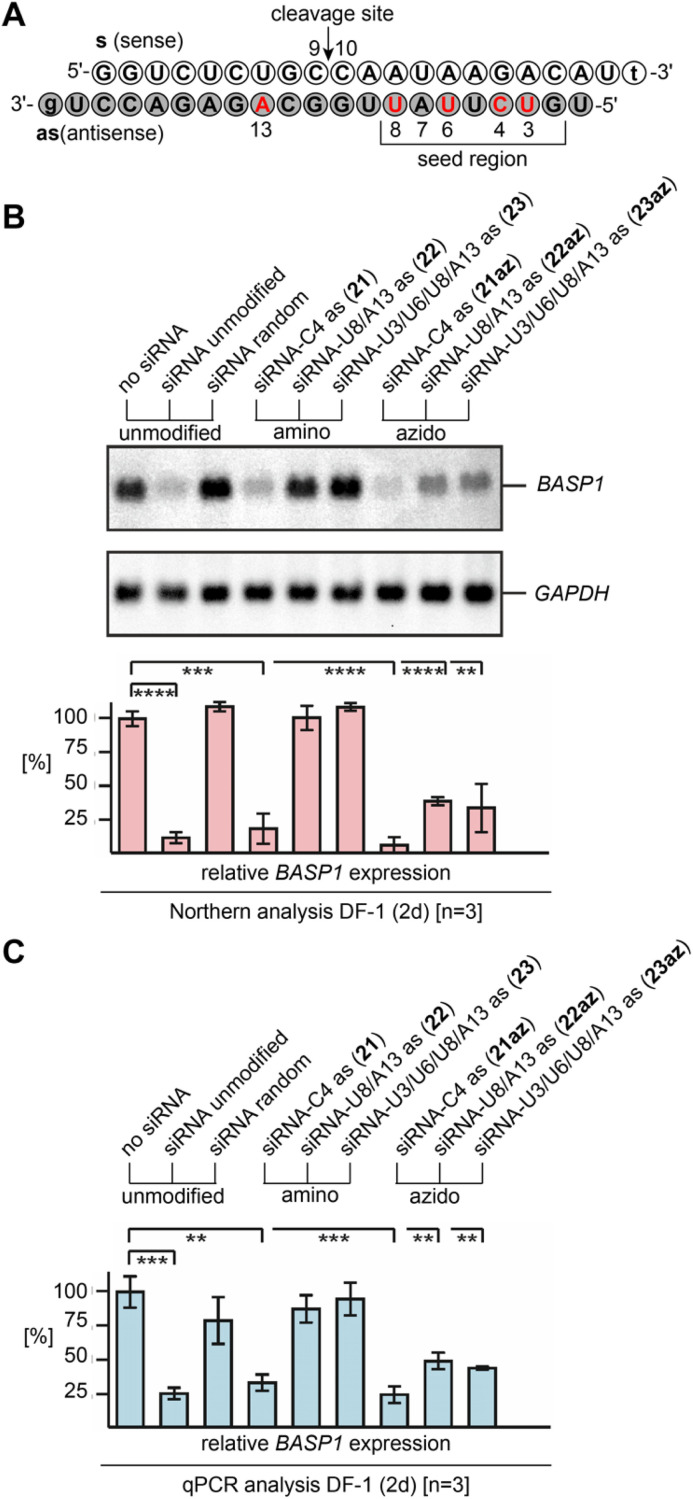
Gene silencing by 2′-NH_2_ and 2′-N_3_ modified siRNAs. (A) Sequence of the brain acid soluble protein 1 (*BASP1*)^44^ targeting siRNA duplex used in this study; nucleosides in red indicate positions for the modification tested. (B) Biological activities of 2′-NH_2_ and 2′-N_3_ modified siRNAs, directed against *BASP1* mRNA. Chicken DF-1 cells grown on 60 mm dishes were transiently nucleofected with 0.24 nmol (∼3.0 μg) aliquots of the individual siRNAs. An equal aliquot of siRNA with a shuffled (random) nucleotide sequence was used as a control. Total RNAs were isolated 2 days after siRNA delivery, and 5 μg of aliquots were analyzed by Northern hybridization using a digoxigenin-labeled DNA probe specific for the chicken *BASP1* gene, and subsequently with a digoxigenin-labeled probe specific for the housekeeping chicken *GAPDH* gene. Sizes for the mRNAs are: *BASP1*, 2.0 kb; *GAPDH*, 1.4 kb. The levels (%) of *BASP1* expression were determined using the program ImageQuant TL and are depicted as bars in relation to mock transfections (no siRNA, 100%). Vertical bars show standard deviations (SD) from independent experiments (*n* = 3). Statistical significance was assessed by using a paired Student *t*-test (**P < 0.01, ***P < 0.001, ****P < 0.0001). (C) The same as (B) but analyzed by quantitative polymerase chain reaction (qPCR) using each 2.5 ng cDNA template reverse transcribed from total RNA, and primers specific for chicken *BASP1* or *GAPDH*. All siRNAs depicted contain overhangs of 2′-deoxynucleosides (lower case letters).

With the new method for efficient 2′-N_3_ RNA synthesis in our hand, we further aimed at demonstrating their siRNA potential by directly comparing with the corresponding 2′-NH_2_ counterparts and by explicitly placing multiple azides into the modification-sensitive seed region of the siRNA antisense strand. Knockdown of the brain acid soluble protein 1 (BASP1)^44^ from the DF-1 chicken cell line by transient siRNA transfection was selected as an established model system (Fig. 5A and ESI Table 2†).^10–12^

The Northern blot and the corresponding graph depicted in Fig. 5B show that the unmodified siRNA efficiently silences the *BASP1* gene, while a control siRNA duplex with two compensatory base pair mutations in the seed region (U7as–A13s and A5as–U15s; siRNA random) did not downregulate.

As expected, a single 2′-NH_2_ or 2′-N_3_ installed at C4 (21, 21az) in the seed region led to efficient silencing. The picture changed when more modifications were present. For the 2′-amino modified siRNAs U8/A13as (22) and U3/U6/U8/A13as (23), the silencing capabilities were abolished. Interestingly, the two corresponding 2′-azido modified siRNAs U8/A13as (22az) and U3/U6/U8/A13 (23az) still exhibited significant silencing activity, which strongly underlines their virtue for siRNA design and performance. The results were independently confirmed by qPCR analysis (Fig. 5C and ESI†).

## Conclusions

2′-Azido RNA has attracted much interest recently because of novel opportunities for the cell and tissue-specific metabolic labeling of nascent RNA. Spitale and co-workers demonstrated the efficient metabolic incorporation of 2′-azidoadenosine into cellular RNA.^45^ 2′-Azidoadenosine is incorporated transcriptionally by RNA polymerase enzymes and post-transcriptionally by poly(A)polymerases. Additionally, through the manipulation of enzymes in the nucleotide salvage pathway, further azido-modified nucleosides have been applied for RNA metabolic labeling. It has been shown that the overexpression of nucleoside kinase UCK2 or deoxynucleoside kinase dCK enables RNA labeling with 2′-azidouridine^46^ and 2′-azidocytidine,^47^ respectively. Moreover, 2′-azidoguanosine has been utilized for AIR-seq (azidonucleoside-incorporated RNA sequencing) which enabled the genome-wide analysis of transcription upon heat stress in *Escherichia coli*, and hence, represents a new benchmark for metabolic RNA labeling to probe RNA dynamics in bacteria.^48^ All these novel developments have significantly increased the demand for site-specifically labeled, chemically synthesized 2′-azido RNAs which are needed to reference the approaches. Additionally, 2′-azido RNA is required for siRNA technologies.

Thus, to satisfy the growing demand, a novel synthetic methodology was conceptualized and diazotransfer reactions on readily accessible 2′-amino RNA with fluorosulfuryl azide (FSO_2_N_3_) were investigated. This reagent was previously introduced for NH_2_-to-N_3_ conversions on small organic compounds,^19,49^ and also used on native RNAs and synthetic RNA–peptide conjugates with structurally well accessible primary amino groups.^20^ For the here demonstrated conversion of the sterically hindered 2′-NH_2_ groups in RNA into their 2′-N_3_ modified counterparts, the diazotransfer reaction had to be carefully optimized, and finally was successful for longer RNA containing multiple 2′-NH_2_ moieties and other sensitive nucleoside modifications, such as 4-thiouridine and 3-methylcytosine. The synthetic ease of generating 2′-azido RNA will pave the way for novel applications in RNA biology, and strengthen existing ones in siRNA technologies and RNA metabolic labeling.

## Conflicts of interest

There are no conflicts to declare.

## Supplementary Material

OB-020-D2OB01560A-s001
