# Supplementary material for: Robust synthesis of 2′-azido modified RNA from 2′-amino precursors by diazotransfer reaction
Source: Org Biomol Chem. 2022 Sep 29;20(39):7845–50. doi: 10.1039/d2ob01560a (PMC9554934; doi:10.1039/d2ob01560a)
Supplement: OB-020-D2OB01560A-s001 [file OB-020-D2OB01560A-s001.pdf]

Electronic Supplementary Material (ESI) for Organic & Biomolecular Chemistry.
This journal is © The Royal Society of Chemistry 2022

Supporting Information

Synthesis of 2'-azido modified RNA from 2'-amino precursors by diazotransfer reaction

Sarah Moreno, José M. Ramos Pittol, Markus Hartl and Ronald Micura

Contents

Supporting Methods

RNA solid-phase synthesis of 2'-NH_2, 4sU and m^3C modified RNA 2
Deprotection and purification of 2'-NH_2 modified RNA 2
Deprotection and purification of 4sU modified RNA 3
Deprotection and purification of m^3C modified RNA 3
Mass spectrometry of oligoribonucleotides 3
Diazotransfer reaction on 2'-NH_2 modified RNA 3
Primer extension analysis 4
RNA interference and analysis of gene silencing 4

Supporting Figures 6

Supporting Figure 1 6
Supporting Figure 2 7
Supporting Figure 3 8
Supporting Figure 4 9
Supporting Figure 5 10
Supporting Figure 6 11
Supporting Figure 7 12
Supporting Figure 8 13

Supporting Tables 14

Supporting Table 1 14
Supporting Table 2 15

References 16

Supporting Methods

RNA solid-phase synthesis 2'-NH_2, 4sU and m^3C modified RNA

Standard phosphoramidite chemistry was applied for RNA strand elongation and incorporation of all 2'-NH_2 modified nucleoside phosphoramidites. *N*-Acetyl-2'-*O*-TOM protected nucleoside phosphoramidite building blocks and 2'-*O*-TBDMS 1000 Å CPG solid support were purchased from ChemGenes. All oligonucleotides were synthesized on an ABI 392 Nucleic Acid Synthesizer following standard methods: detritylation (90 sec) with dichloroacetic acid/1,2-dichloroethane (4/96); coupling (5.0 min; 10 min for 2-NH_2-G building block) with phosphoramidites/ acetonitrile (100 mM, 200 µL) and 5-(benzylthio)-1*H*-tetrazol/acetonitrile (300 mM, 500 µL); capping (2x 25 sec) with Cap A/Cap B (1/1) for unmodified and modified RNA, Cap A: 4-(dimethylamino)pyridine/acetonitrile (500 mM), Cap B: acetic anhydride/*sym*-collidine/acetonitrile (2/3/5); oxidation (1 min) with iodine (20 mM) in tetrahydrofuran/pyridine/H_2O (35/10/5). Solutions of phosphoramidites, tetrazole and Cap A were dried over activated molecular sieves (3 Å) overnight.

Deprotection and purification of 2'-NH_2 modified RNA

For deprotection of unmodified and 2'- NH_2 modified RNA, the solid support was mixed with aqueous methylamine (40%, 0.50 ml) and aqueous ammonia (28%, 0.50 ml) for 30 minutes at 65°C. The supernatant was removed and the solid support was washed trice with H_2O/tetrahydrofuran (1.0 ml; 1/1). Combined supernatant and washings were evaporated to dryness and the residue was dissolved in a solution of tetra-*n*-butylammonium fluoride in tetrahydrofuran (1.0 M, 1.0 ml) and incubated for 14 h at 37 °C for removal of 2'-*O*-silyl protecting groups. The reaction was quenched by addition of triethylammonium acetate/H_2O (1.0 M, 1.5 ml, pH 7.4). Tetrahydrofuran was removed under reduced pressure and the sample was desalted with size-exclusion column chromatography (GE Healthcare, HiPrep™ 26/10 Desalting; Sephadex G25) eluting with H_2O; collected fractions were evaporated and the RNA dissolved in H_2O (1 ml). The crude RNA was purified by anion exchange chromatography (GE Healthcare Äkta Basic HPLC System) on a semipreparative Dionex DNAPac® PA-100 column (9 mm x 250 mm) at 60°C with a flow rate of 2 ml/min (eluent A: 20 mM $NaClO_4$, 25 mM Tris·HCl, pH 8.0, 20 v/v % acetonitrile; eluent B: 600 mM $NaClO_4$, 25 mM Tris·HCl, pH 8.0, 20% v/v acetonitrile). Fractions containing RNA were evaporated and the residue redissolved in 0.1 M triethylammonium bicarbonate solution (10 to 20 ml), loaded on a C18 SepPak Plus® cartridge (Waters/Millipore), washed with H_2O, and then eluted with acetonitrile/H_2O (1/1). Crude and purified RNA were analyzed by anion exchange chromatography (GE Healthcare Äkta Basic HPLC System) on a Dionex DNAPac® PA-100 column (4 mm × 250 mm) at 60 °C with a flow rate of 1 ml/min. Gradients up to 60% B in A were applied; eluent A: 20 mM $NaClO_4$, 25 mM

Tris·HCl, pH 8.0, 20% v/v acetonitrile; eluent B: 600 mM $NaClO_4$, 25 mM Tris·HCl, pH 8.0, 20% v/v acetonitrile. HPLC traces were recorded at UV absorption by 260 nm. RNA quantification was performed on an Implen P300 Nanophotometer.

Deprotection and purification of 4sU modified RNA[1,2]

After synthesis, the solid support was treated with 1,8-diazabicyclo[5.4.0]undec-7-en (DBU) in anhydrous acetonitrile (5 mL, 1 M) for two hours at room temperature and washed with dry acetonitrile (2x 1 mL mL) to remove residual DBU. The solid support was then suspended in a mixture of aqueous methylamine (40%, 0.50 ml) and aqueous ammonia (28%, 0.50 ml) for 30 minutes at 65 °C additionally containing 1,4-dithiothreitol (50 mM). After filtration and evaporation, desilylation was carried out as described above.

Deprotection and purification of m^3C modified RNA[3]

For basic deprotection of m^3C modified RNA, the solid support was treated with a mixture of aqueous ammonia (28-30%, 0.50 mL) and ethanol (0.50 mL) for 4 hours at 50 °C. Further proceedings are identical to the ones described for 2'-NH_2-modified RNA.

Mass spectrometry of oligoribonucleotides

RNA samples (~200 pmol in ~3 µl) were diluted with aqueous solution of ethylenediaminetetraacetic acid disodium salt dihydrate (Na_2H_2EDTA, 40 mM, 15 µl). Water was added to obtain a total volume of 30 µl. The sample injected onto a C18 XBridge column (2.5 µm, 2.1 mm × 50 mm) at a flow rate of 0.1 ml/min and eluted using a gradient 0 to 100% B gradient at 30°C (eluent A: 8.6 mM triethylamine, 100 mM 1,1,1,3,3,3-hexafluoroisopropanol in H_2O; eluent B: methanol). RNA was detected by a Finnigan LCQ Advantage Max electrospray ionization mass spectrometer with 4.0 kV spray voltage in negative mode.

Diazotransfer reaction on 2'-NH_2 modified RNA

2'-NH_2 modified RNA (2 to 10 nmol) was lyophilized and dissolved in 100 µl $NaHCO_3$ buffer (0.1 M, pH 8.22) and 20 µl DMF. Fluorosulfuryl azide (FSO_2N_3, 150 mM) in methyl *tert*-butyl ether (MTBE) was prepared according to Krasheninina *et al.* (see ref. [4]) and 100 µl of a 150 mM FSO_2N_3 solution were added to the reaction mixture that was tightly sealed. Samples were incubated for 24 h at 25 °C and 1500 rpm. Separation of the phases was facilitated by centrifugation (10 min, 13000 rpm) and the upper organic layer was discarded. RNA was precipitated by the addition acetone (1 ml) containing 2% sodium

perchlorate. The RNA was stored at -20 °C for at least 30 min and was then isolated by centrifugation (4 °C, 30 min, 13000 rpm). The pellet was washed twice with acetone (600 ml each), was air dried and was then analyzed by AE-HPLC and LC-MS.

Primer extension assay

The 37 nt RNA 5′-AUUCCUCXUCAUCCAUACAGACAGAACUAACGAUUCG (10 µl; 1 µM) and 4 µl of Alexa Fluor 647 5′-end labelled DNA primer (2 pmol/µl; IDT; 5′-/5Alexa647N/CGAATCGTTAGTTCTGTC-3′) were annealed for 5 min at 65 °C, then incubated at 35 °C for 5 min and cooled on ice for 1 min (performed in an Eppendorf Mastercycler personal). Then, 8 µl of a 'primer extension mix' containing 4 µl of 5× SS IV RT buffer (provided by supplier), 1 µl of 0.1 M 1,4-dithiothreitol (DTT) solution, 1 µl of 5 mM dNTPs mixture (1.25 mM for each dNTP), 2 µl of dimethyl sulfoxide (DMSO), and 0.4 µl of Super-Script IV reverse transcriptase (200 U/µl; Invitrogen) were added and the reaction mixtures incubated at 55 °C for 10 min.[5,6] The primer extension reaction was stopped by addition of 1 µl of 4 M NaOH solution, the mixture was incubated at 90 °C for 5 min, and then cooled on ice. The Alexa Fluor 647 labelled cDNA strands were precipitated by adding 90 µl of precipitation solution (650 µl water, 150 µl 1 M NaOAc pH 5.2, 10 µl of 20 mg/ml glycogen) and 250 µl of cold ethanol and stored for 30 min at –20 °C. After centrifugation for 30 min at 4 °C at 11740 rpm, the supernatant was removed and the pellets dried under reduced pressure. The pellets were resuspended in 8 µl of gel loading buffer (97% formamide, 10 mM EDTA), loaded on 10% polyacrylamide gels with 7 M urea and run for approximately 120 min at 35 W. Sequencing ladders were produced by adding 2 µl of 5 mM ddNTPs to the RNA samples prior to addition of the 'primer extension mix'. The extension products were analyzed by scanning the gel at 635 nm with a Typhoon FLA 9500 instrument (GE Healthcare).

RNA interference and analysis of gene silencing

Lyophilized synthetic siRNA duplexes were dissolved, annealed, and delivered into DF-1 cells by electroporation as described previously.[7] Total RNA isolation, gel electrophoresis, and Northern blotting was done as described.[7] To prepare digoxigenin (DIG)-dUTP labeled probes specific for chicken *BASP1* or *GAPDH*, 300-bp segments from the respective coding regions were amplified by polymerase chain reaction (PCR) using the PCR DIG Probe Synthesis Kit (Roche, Cat. No. 11636090910), and *BASP1* (5'-GTTGAGAAGGATGCTCAGGTC-3'/5'-TTTGCTCTTGTCATCTGCTTTG-3') or *GAPDH* (5'-GCAGGTGCTGAGTATGTTG-3'/5'-ATCCACCGTCTTCTGTG-TG-3') specific primer pairs. PCR was performed with 30 cycles (95°C, 30 s; 50°C, 30 s, 72°C, 40 s) using 2 ng of DNA template obtained from a preceding PCR performed under standard conditions. The total yield of the DIG-labeled probes was 4 µg each. Filters were hybridized at 40 °C as described in ref. [5] in a buffer containing 50% (v/v) formamide, 5x SSC, 0.1%

(w/v) lauroylsarcosine, 0.02% (w/v) SDS, and 2% (v/v) Blocking Solution (Roche, Cat. No. 11585762001). After prehybridization for 24 h, filters were hybridized for another 24 h in presence of the DIG-labeled probes (50 ng/ml). Filters were finally washed in 0.2x SSC, 0.1% (w/v) SDS at 60 °C. Afterwards, filters were rinsed in Washing Solution (Roche, Cat. No. 11585762001), and then incubated for 2 h in 1% (w/v) Blocking Reagent (Roche, Cat. No. 11363514910). Filters were subsequently incubated for 60 min in 1% (w/v) Blocking Reagent containing 1:10,000 diluted anti-Digoxigenin-AP (75 mU/µl) (Roche, Cat. No. 11363514910), and then washed three times in Washing Solution. Alkaline phosphatase (AP) activity was detected in a buffer containing 0.1 M NaCl, 0.1 M Tris-HCl pH 9.5 and the 1:100 diluted chemoluminescent substrate disodium 3-(4-methoxy-spiro{1,2-dioxetane-3,2'-(5'-chloro) tricyclo [3.3.1.13,7]decan}-4-yl) phenyl phosphate (CSPD) (Roche, Cat. No. 11363514910). Signals were detected on a Fusion FX imaging system (Vilber) with an exposure time of 10 min, and quantified using the program ImageQuant TL (GE Healthcare).

Quantitative PCR (qPCR) was performed as described in ref. [8] using specific chicken *BASP1* (5'-ACTTTCTGCCAACTTGACAC-3'/5'-TCACTCCCAAACCCCATTAC-3') and *GAPDH* (5'-TCTTCACCACCGCTCAGT-TC-3'/5'-TCAGTTTCTATCAGCCTCTCCC-3') primer pairs. qPCR was performed on a Step One Real-Time PCR System (Applied Biosystems) with 45 cycles (95°C, 15 s; 60°C, 30 s) using 2.5 ng of cDNA template and the Luna® Universal qPCR Master kit (New England BioLabs). Melting curve analyses of the PCR products were performed at the end of each run. The cDNA templates were generated by reverse transcription using the LunaScript® RT SuperMix kit (New England BioLabs). Thereby, each 1 µg of total RNA was mixed with random hexamer and oligo-dT primers, dNTPs, murine RNase inhibitor, and Luna® reverse transcriptase in a final volume of 20 µl. The reaction was incubated in a thermocycler for 2 min at 25 °C, 10 min at 55 °C, 1 min at 95 °C, and then cooled down to 4 °C. Quantification of the signals was performed using the OneStep software (Applied Biosystems).

Supporting Figures

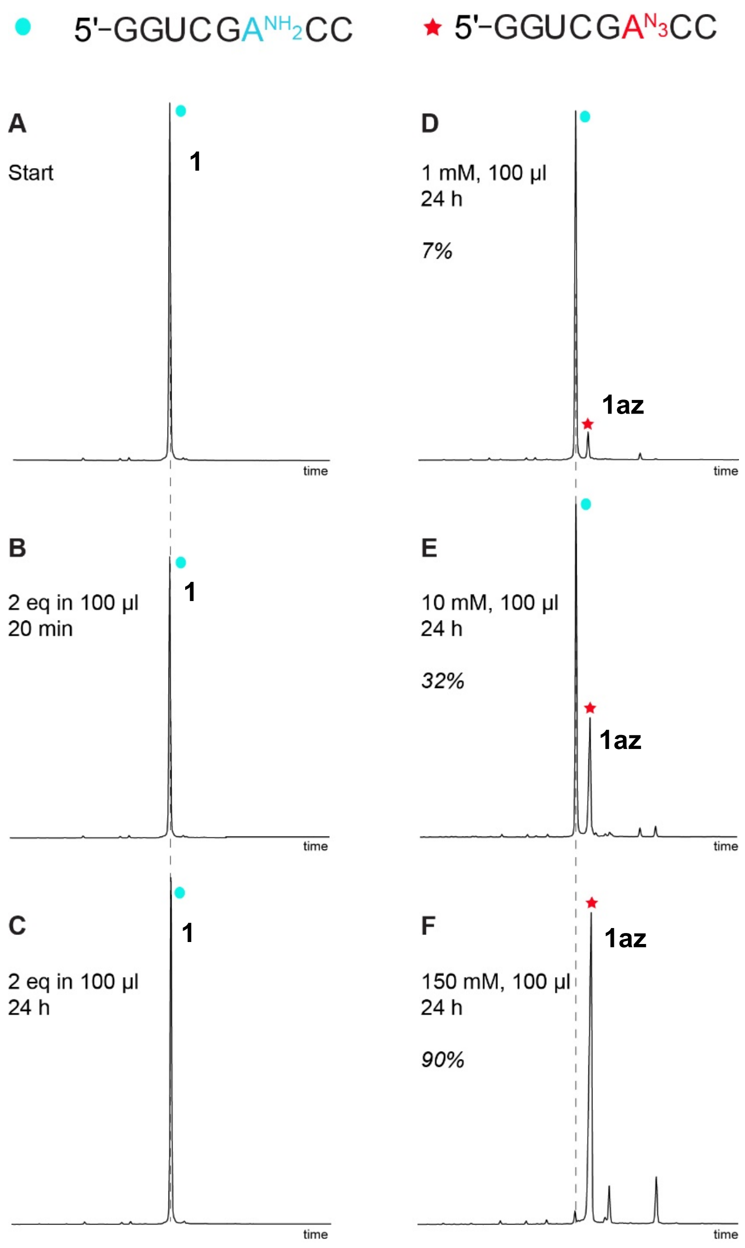

Supporting Figure 1. Evaluation of the concentration of fluorosulfuryl azide (FSO_2N_3) for the diazotransfer reaction on 2'-amino modified RNA. **A)** AE-HPLC of the RNA starting material; **B)** 2 nmol RNA in 100 µl 100 mM $NaHCO_3$ (pH 8.22), 20 µl DMF, 2 eq FSO_2N_3 in 100 µl MTBE, 1500 rpm, 20 min, precipitation, AE-HPLC; **C)** 2 eq FSO_2N_3 in 100 µl MTBE, 24 h; **D)** 1 mM FSO_2N_3 in 100 µl MTBE, 24 h, 7%; **E)** 10 mM FSO_2N_3 in 100 µl MTBE, 24 h, 32%; **F)** 150 mM FSO_2N_3 in 100 µl MTBE, 24 h, 90%. Yields were determined from areas in the HPLC chromatograms relative to the sum of all other peaks.

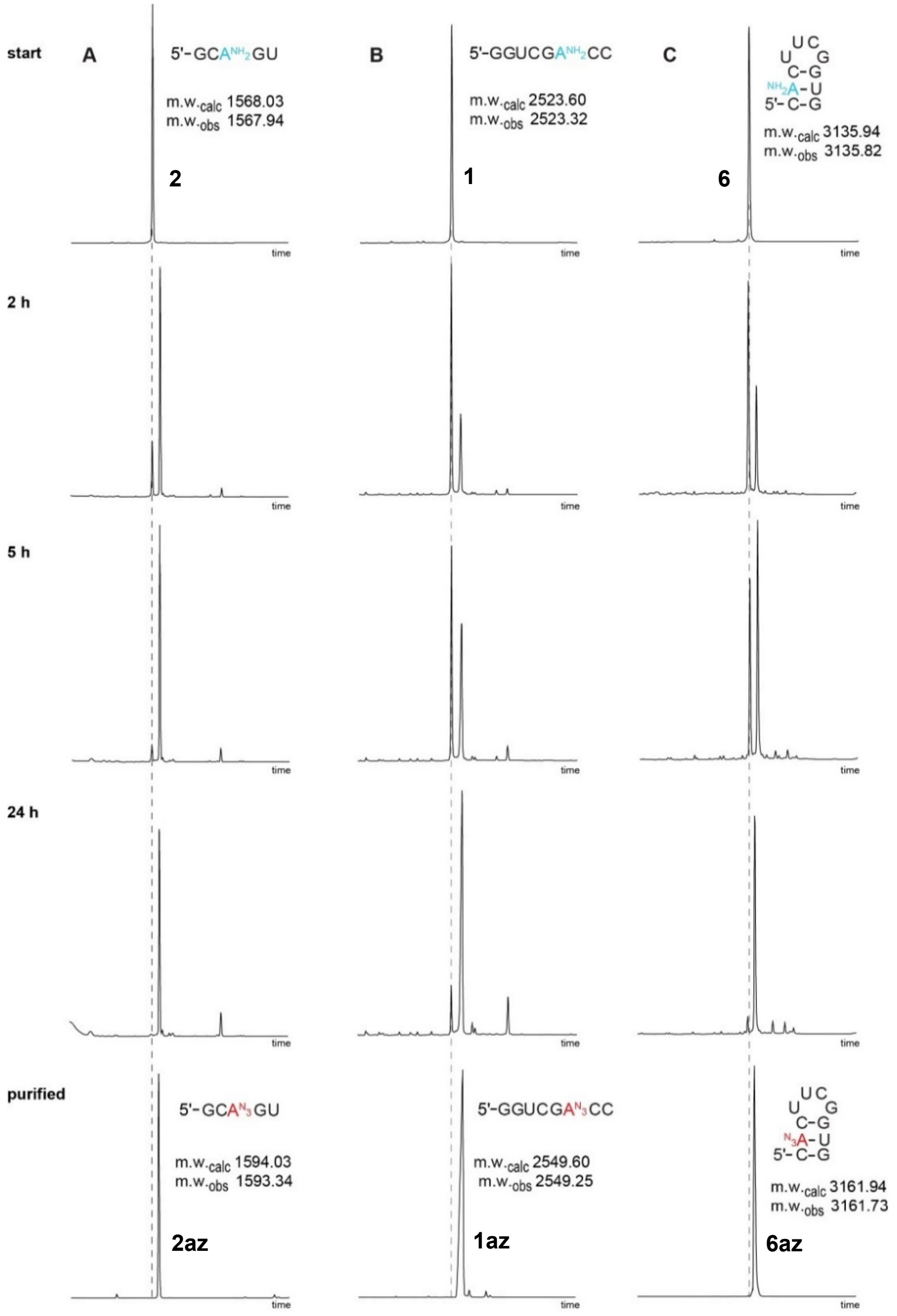

Supporting Figure 2. Diazotransfer on 2'-amino-adenosine modified RNA. **A)** Short 5 nt long RNA sequence without secondary structure; **B)** 8 nt long palindrome; **C)** 10 nt long hairpin. Reaction progress was monitored by anion-exchange HPLC after 2, 5 and 24 h. The 5 nt long RNA exhibits clearly the fastest turnover followed by the 10 nt hairpin and the 8 nt palindrome.

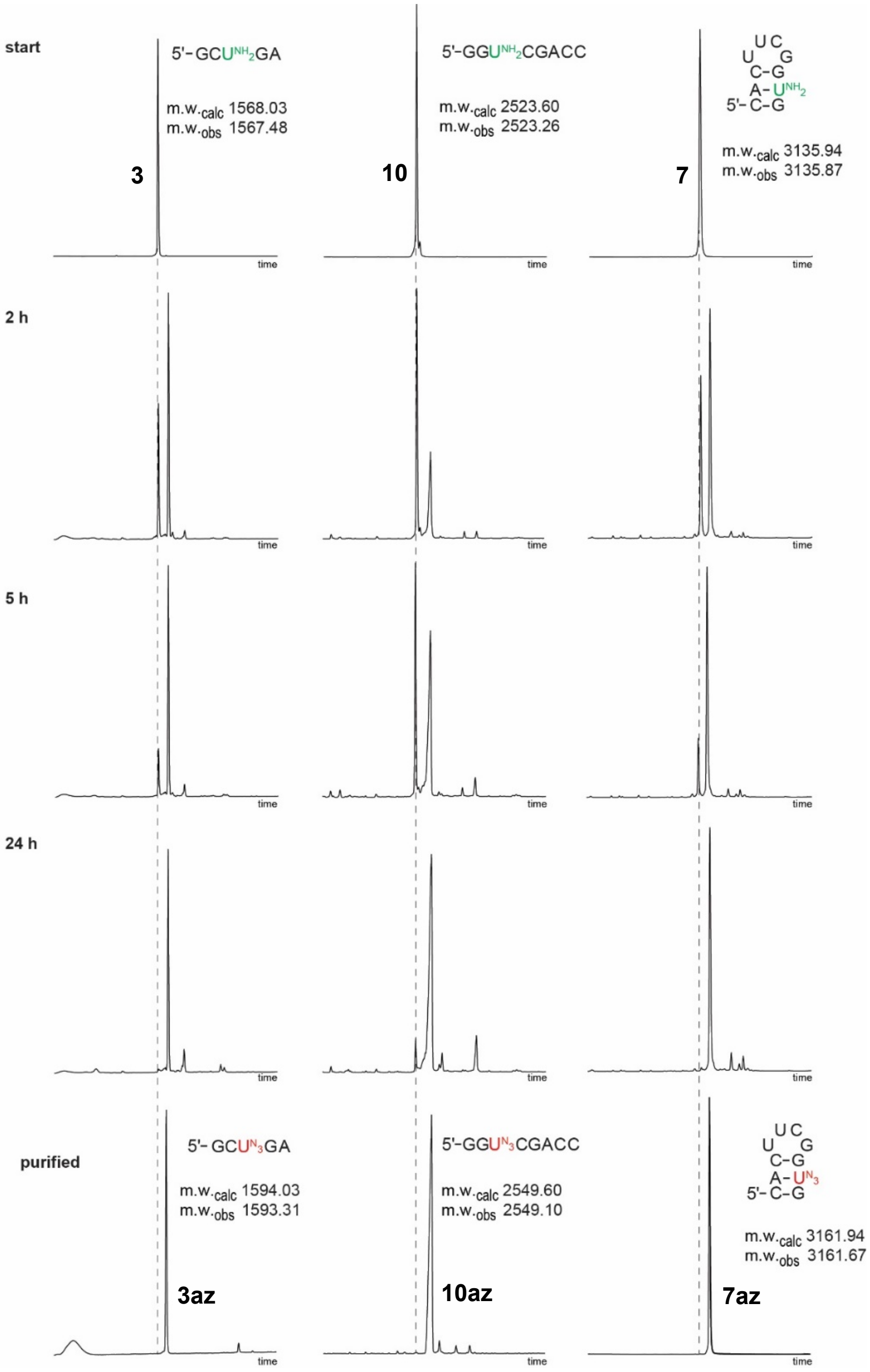

Supporting Figure 3. Diazotransfer on 2'-amino-uridine modified RNA. **A)** Short 5 nt long RNA without secondary structure; **B)** 8 nt long palindrome; **C)** 10 nt long hairpin. Reaction progress was monitored by anion-exchange chromatography after 2 h, 5 h and 24 h. The 5 nt long RNA exhibits clearly the fastest turnover followed by the 10 nt hairpin and the 8nt palindrome.

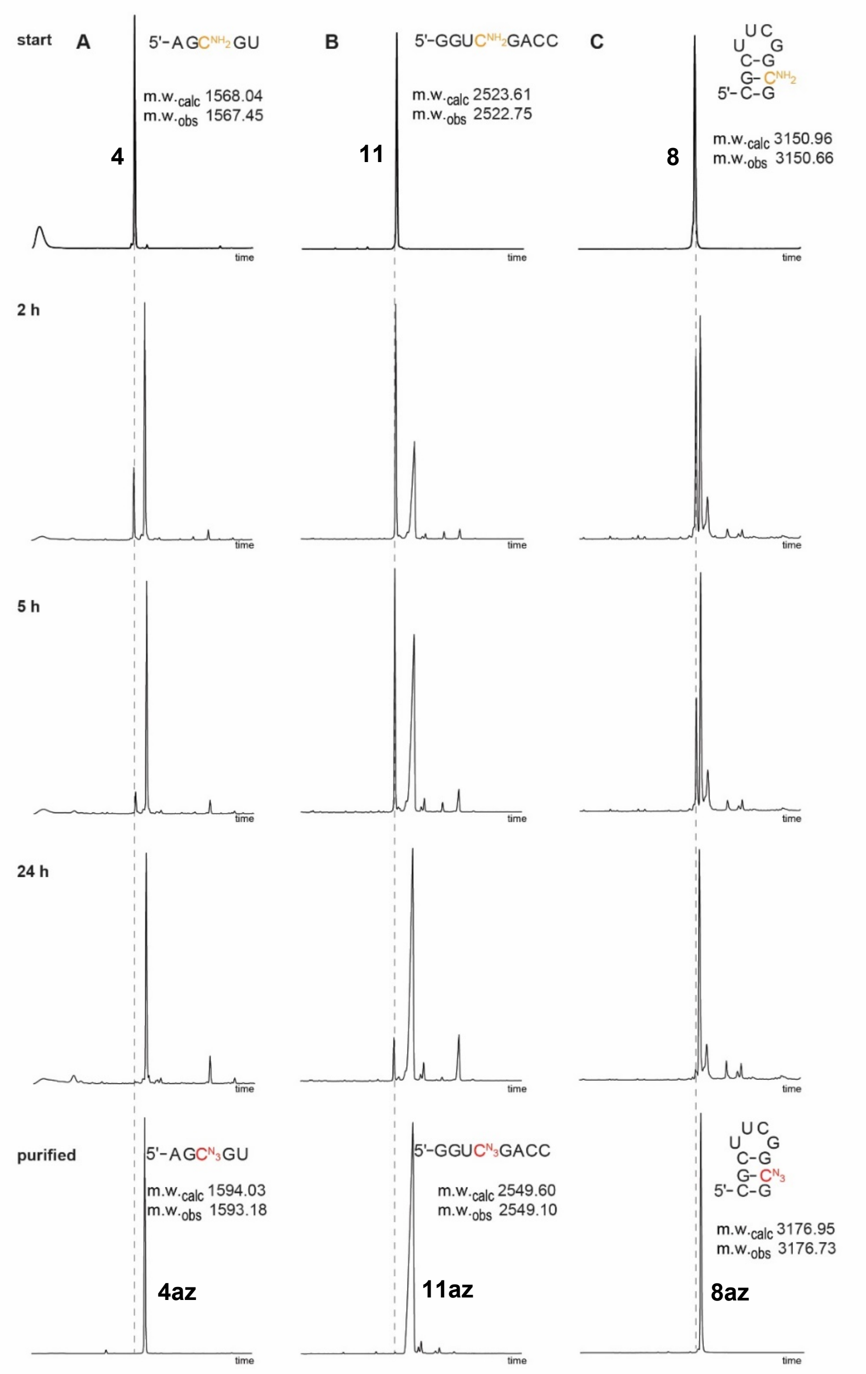

Supporting Figure 4. Diazotransfer on 2´-amino-cytidine modified RNA **A)** Short 5 nt long RNA sequence without secondary structure **B)** 8 nt long palindrome **C)** 10 nt long hairpin. Reaction progress was monitored by anion-exchange chromatography after 2 h, 5 h and 24 h. 5 nt long sequence exhibits clearly the fastest turnover followed by the 10 nt hairpin and the 8nt palindrome.

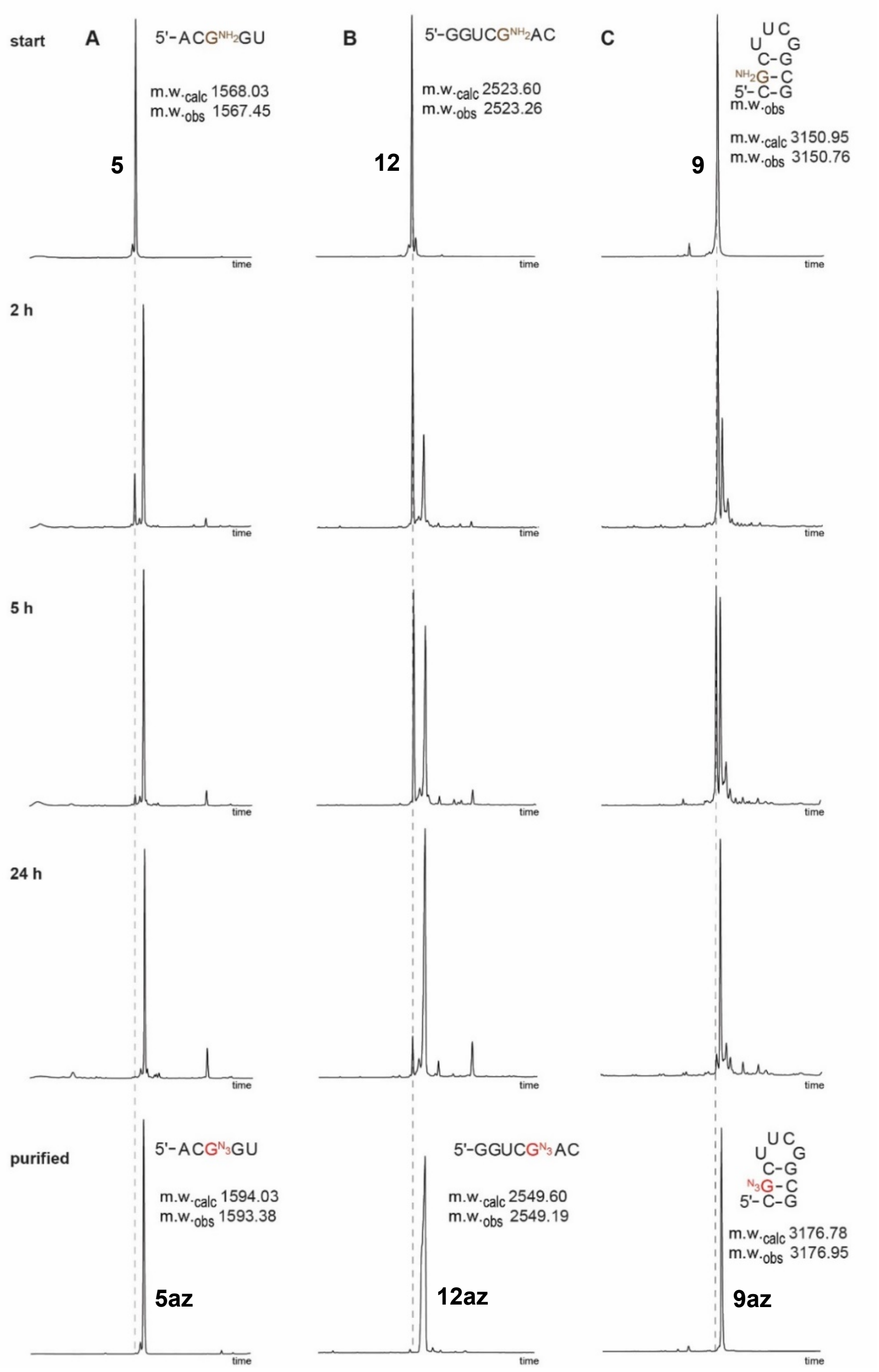

Supporting Figure 5. Diazotransfer on 2'-amino-guanosine modified RNA. **A)** Short 5 nt long RNA without secondary structure; **B)** 8 nt long palindrome; **C)** 10 nt long hairpin. Reaction progress was monitored by anion-exchange chromatography after 2 h, 5 h and 24 h. The 5 nt long RNA exhibits clearly the fastest turnover followed by the 10 nt hairpin and the 8 nt palindrome.

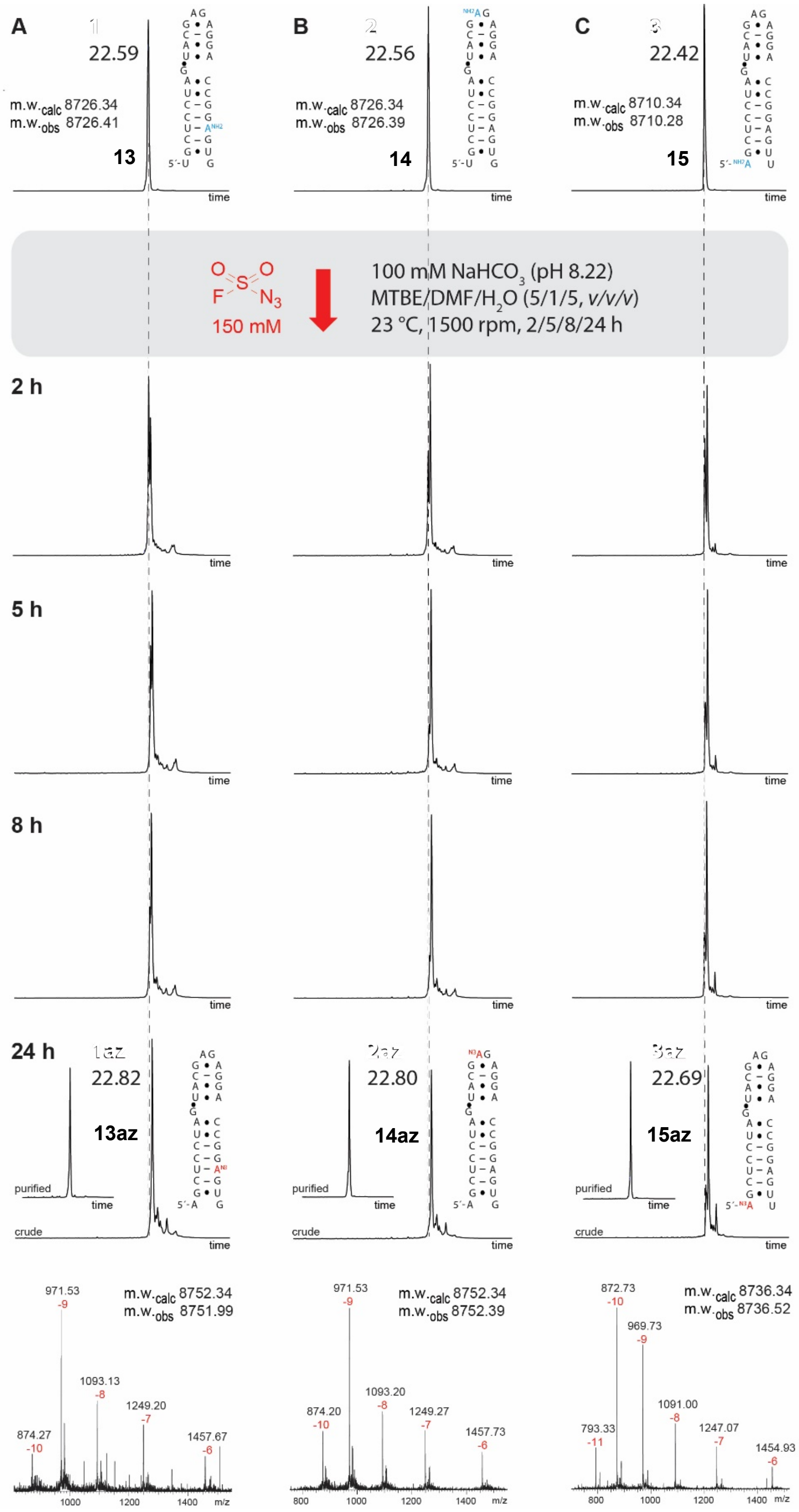

Supporting Figure 6. Sarcin-ricin loop RNA containing 2'-amino-adenosine **A)** in the stem region; **B)** in the loop region; and **C)** at the 5'-terminus. RNAs were treated with 150 mM fluorosulfuryl azide in MTBE/DMF/H_2O containing 100 mM $NaHCO_3$. Reaction progress was monitored by anion-exchange chromatography at the time points indicated. Converted RNAs were purified and molecular weights were confirmed by LC-ESI mass spectrometry.

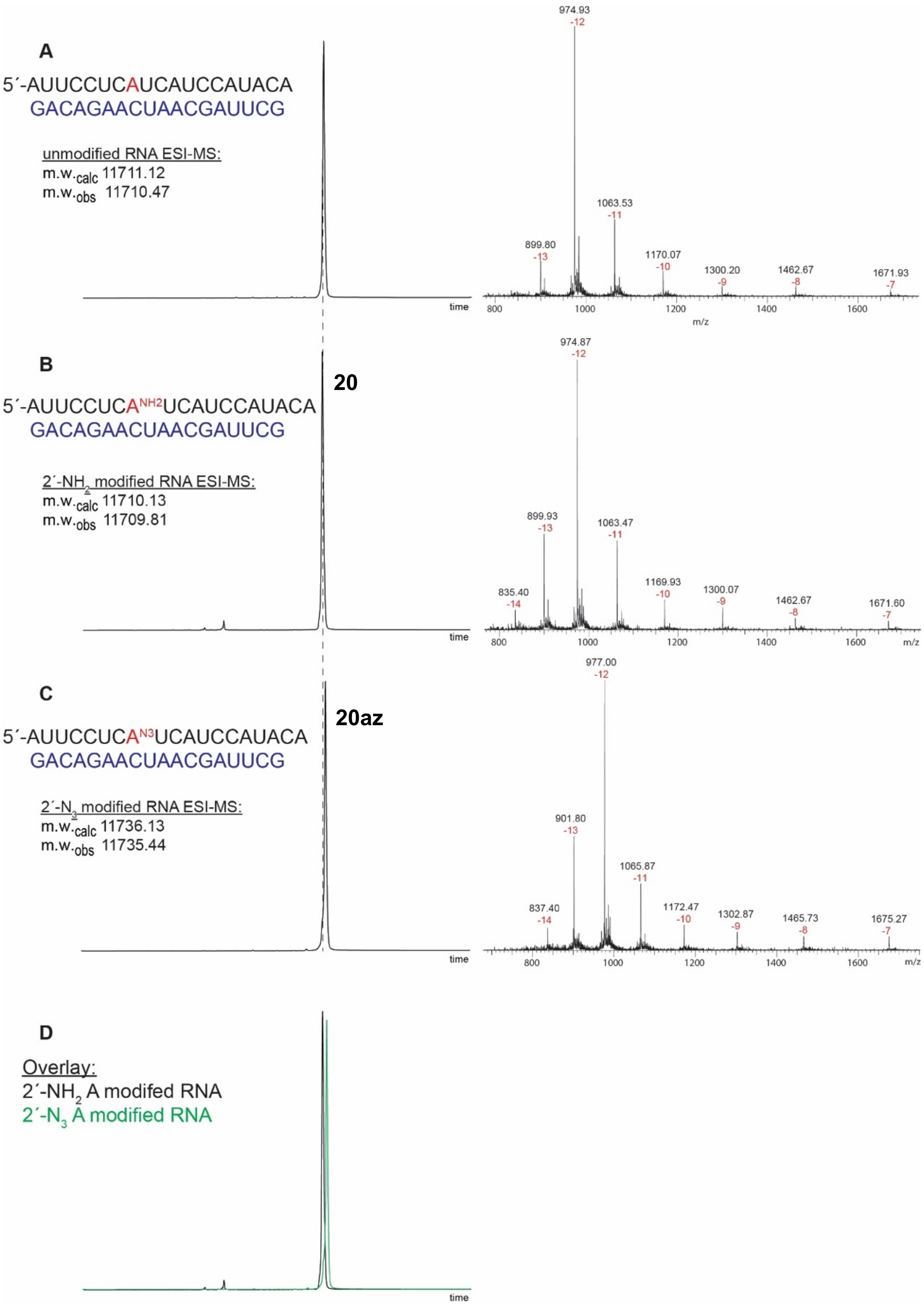

Supporting Figure 7. RNA templates for primer extension analysis. **A)** Unmodified RNA; **B)** 2'-amino adenosine modified RNA; **C)** purified 2'-azido RNA with corresponding LC-ESI mass spectra. **D)** Overlay of the 2'-amino (black) and 2'-azido (green) modified RNA.

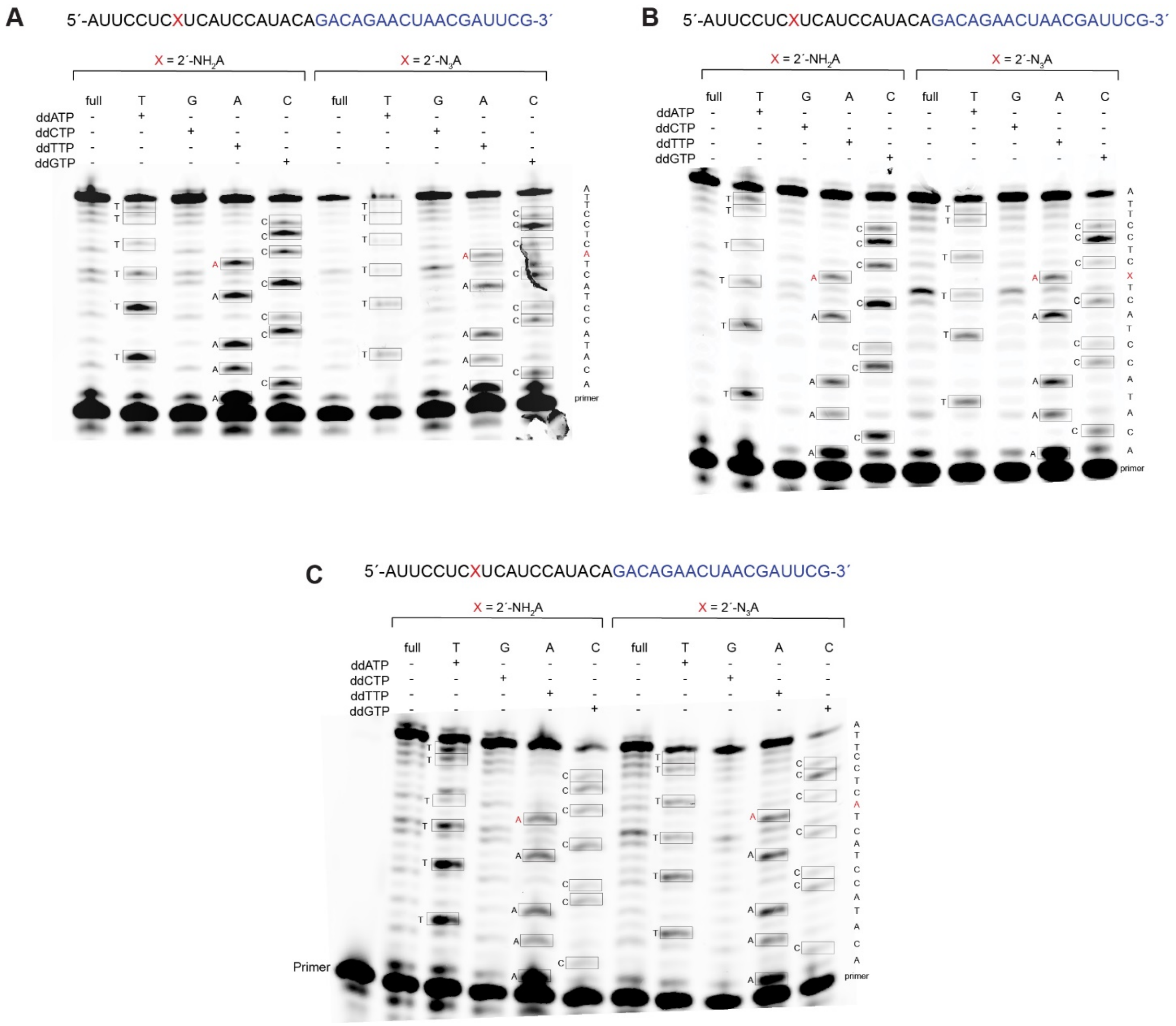

Supporting Figure 8. Three replicates of the primer extension assay for 2'-amino-A and 2'-azido-A modified RNAs (**20**, **20az**).

Table S1. List of 2'-amino and 2'-azido-modified RNAs synthesized in this study.

No.	Sequence	m.w.^{calc}	m.w.^{obs}
**1**	GGUCG(A^{NH2})CC	2523.60	2523.32
**1az**	GGUCG(A^{N3})CC	2549.60	2549.25
**2**	GC(A^{NH2})GU	1568.03	1567.94
**2az**	GC(A^{N3})GU	1594.03	1593.34
**3**	GC(U^{NH2})GA	1568.03	1567.48
**3az**	GC(U^{N3})GA	1594.03	1593.31
**4**	AG(C^{NH2})GU	1568.04	1567.45
**4az**	AG(C^{N3})GU	1594.03	1593.18
**5**	AC(G^{NH2})GU	1568.03	1567.45
**5az**	AC(G^{N3})GU	1594.03	1593.38
**6**	C(A^{NH2})CUUCGGUG	3135.94	3135.82
**6az**	C(A^{N3})CUUCGGUG	3161.94	3161.73
**7**	CACUUCGG(U^{NH2})G	3135.94	3135.87
**7az**	CACUUCGG(U^{N3})G	3161.94	3161.67
**8**	CGCUUCGG(C^{NH2})G	3150.96	3150.66
**8az**	CGCUUCGG(C^{N3})G	3176.95	3176.73
**9**	C(G^{NH2})CUUCGGCG	3150.95	3150.76
**9az**	C(G^{N3})CUUCGGCG	3176.78	3176.95
**10**	GG(U^{NH2})CGACC	2523.60	2523.26
**10az**	GG(U^{N3})CGACC	2549.60	2549.10
**11**	GGU(C^{NH2})GACC	2523.61	2522.75
**11az**	GGU(C^{N3})GACC	2549.60	2549.10
**12**	GGUC(G^{NH2})ACC	2523.60	2523.26
**12az**	GGUC(G^{N3})ACC	2549.60	2549.19
**13**	UGCUCCUAGUACGAGAGGACCGG(A^{NH2})GUG	8726.34	8726.41
**13a**	UGCUCCUAGUACGAGAGGACCGG(A^{N3})GUG	8752.34	8751.99
**14**	UGCUCCUAGUACG(A^{NH2})GAGGACCGGAGUG	8726.34	8726.39
**14a**	UGCUCCUAGUACG(A^{N3})GAGGACCGGAGUG	8752.34	8752.39
**15**	(A^{NH2})GCUCCUAGUACGA)GAGGACCGGAGUG	8710.34	8710.28
**15a**	(A^{N3})GCUCCUAGUACGAGAGGACCGGAGUG	8736.34	8736.52
**16**	G(C^{NH2})GG(A^{NH2})U(U^{NH2})C(G^{NH2})C	-	-
**16az**	G(C^{N3})GG(A^{N3})U(U^{N3})C(G^{N3})C	3276.02	3275.27
**17**	C($^{2'ap}U$)CUUCGG(A^{NH2})G	-	-
**17az**	C($^{2'ap}U$)CUUCGG(A^{N3})G	3244.93	3244.78
**18**	CUC(^{4s}U)UCGG(A^{NH2})G	-	-
**18az**	CUC(^{4s}U)UCGG(A^{N3})G	3178.00	3177.31
**19**	CU(^{m3}C)UU(C^{NH2})GGUG	-	-
**19az**	CU(^{m3}C)UU(C^{N3})GGUG	3175.97	3175.31
**20**	AUUCCUC(A^{NH2})UCAUCCAUACAGACAGAACUAACGAUUCG	11710.13	11709.81
**20az**	AUUCCUC(A^{N3})UCAUCCAUACAGACAGAACUAACGAUUCG	11736.13	11735.44

Table S2. List of 2'-amino and 2'-azido-modified siRNA duplexes used in this study.

No.	siRNA sense and antisense sequences	
**21**	5′-GGUCUCUGCCAAUAAG---ACAUT-3'	sense
	3'-dGUCCAGAGACGGUUAUUC^{NH2}UGU-5'	antisense
**21az**	5′-GGUCUCUGCCAAUAAG--ACAUT	sense
	3'-dGUCCAGAGACGGUUAUUC^{N3}UGU-5'	antisense
**22**	5′-GGUCUCU---GCCAA---UAAGACAUT	sense
	3'-dGUCCAGAGA^{NH2}CGGUU^{NH2}AUUCUGU-5'	antisense
**22az**	5′-GGUCUCU--GCCAA--UAAGACAUT	sense
	3'-dGUCCAGAGA^{N3}CGGUU^{N3}AUUCUGU-5'	antisense
**23**	5′-GGUCUCU---GCCAA---UA---AGA---CAUT	sense
	3'-dGUCCAGAGA^{NH2}CGGUU^{NH2}AU^{NH2}UCU^{NH2}GU-5'	antisense
**23az**	5′-GGUCUCU--GCCAA--UA--AGA--CAUT	sense
	3'-dGUCCAGAGA^{N3}CGGUU^{N3}AU^{N3}UCU^{N3}GU-5'	antisense

References

[1] C. Riml, T. Amort, D. Rieder, C. Gasser, A. Lusser and R. Micura, *Angew. Chem. In. Ed.*, 2017, **56**, 13479.

[2] C. Gasser, I. Delazer, E. Neuner, K. Pascher, K. Brillet, S. Klotz, L. Trixl, M. Himmelstoß, E. Ennifar, D. Rieder, A. Lusser and R. Micura, *Angew. Chem. In. Ed.*, 2020, **59**, 6881.

[3] S. Moreno, L. Flemmich and R. Micura, *Monatsh. Chem. – Chem. Month.*, 2022, **153**, 285.

[4] O. A. Krasheninina, J. Thaler, M. D. Erlacher and R. Micura, *Angew. Chem. In. Ed.*, 2021, **60**, 6970.

[5] S. Lusvarghi, J. Sztuba-Solinska, K. J. Purzycka, J. W. Rausch, and S. F. Le Grice, *J. Vis. Exp*., 2013, **75**, e50243.

[6] C. Gasser, J. Gebetsberger, M. Gebetsberger and R. Micura, *Nucleic Acids Res*., 2018, **46**, 6983–6995.

[7] M. Aigner, M. Hartl, K. Fauster, J. Steger, K. Bister, R. Micura, *Chembiochem*, 2011, **12**, 47-51.

[8] T. Valovka, M. Schönfeld, P. Raffeiner, K. Breuker, T. Dunzendorfer-Matt, M. Hartl, K. Bister, *Sci. Rep.*, 2013, **3**, 3444.
